# A Retrospective Real-World Multicenter Study of the Efficacy and Safety of Desidustat in Chronic Kidney Disease-Related Anemia

**DOI:** 10.7759/cureus.109574

**Published:** 2026-05-24

**Authors:** Sanjeev Gulati, Dilip Pahari, Karan Saraf, Rohit Rungta, V K Sinha, Prashant Bendre, Kaushal Joshi

**Affiliations:** 1 Nephrology, Fortis Escorts, Okhla, IND; 2 Nephrology and Transplant, Medica Superspeciality Hospital, Kolkata, IND; 3 Nephrology, Apollo Excelcare, Guwahati, IND; 4 Nephrology, Medica Superspeciality Hospital, Kolkata, IND; 5 Nephrology, Max Super Speciality Hospital, Noida, IND; 6 Nephrology, Dr. Bendre Kidney Center, Meerut, IND; 7 Medical Affairs, Zydus Nephrology, Zydus Lifesciences Limited, Ahmedabad, IND

**Keywords:** chronic kidney disease (ckd), ckd-related anemia, desidustat, hypoxia-inducible factor, prolyl hydroxylase inhibitor (hif-phi)

## Abstract

Background: Chronic kidney disease (CKD) often leads to a relative deficiency of erythropoietin (EPO), resulting in anemia. Exogenous erythropoietin analogs are commonly used to address this deficiency. Desidustat, a recently approved hypoxia-inducible factor prolyl hydroxylase inhibitor (HIF-PHI), enhances endogenous EPO production. This study evaluates the multicenter outcomes of desidustat in treating CKD-induced anemia.

Methodology: This retrospective analysis included patients with CKD-induced anemia who received desidustat. Patients were followed up at one month, two months, and six months post-treatment initiation. Oral or intravenous (IV) iron was administered based on iron depletion status. Dosage adjustments, therapy compliance, hemoglobin (Hb) levels, and safety parameters were assessed at each follow-up.

Results: The study included 517 patients with CKD-induced anemia, comprising 39.38% (204) male patients and 60.61% (313) female patients, with a mean age of 57.86 ± 14.24 years. Comorbid conditions included diabetes mellitus (44.29%, 229 patients) and hypertension (61.31%, 317 patients). The majority of patients (88.18%, 456 patients) received desidustat 100 mg thrice weekly. Hemoglobin levels increased from a baseline mean of 9.11 ± 1.38 g/dL to 9.83 ± 1.52 g/dL at follow-up 1, 10.10 ± 1.59 g/dL at follow-up 2, and 10.13 ± 1.74 g/dL at follow-up 3 (p < 0.005), reflecting sustained improvements of 9.13% (0.72 g/dL), 12.05% (0.99 g/dL), and 12.39% (1.02 g/dL) from baseline, respectively. Both dialysis-dependent and pre-dialysis patients showed a consistent rise in Hb levels from baseline, with a numerically greater increase observed in the pre-dialysis group, likely due to better residual kidney function. The treatment was generally well-tolerated, with mild adverse events (AEs) such as constipation and throat pain, which did not require discontinuation of therapy.

Conclusion: Desidustat is a safe and effective oral therapy for CKD-induced anemia, demonstrating consistent Hb increases in both dialysis and pre-dialysis patients. Its oral administration route enhances usability, particularly in tropical conditions.

## Introduction

Anemia is a common and clinically significant complication of chronic kidney disease (CKD), and its frequency tends to increase with worsening renal dysfunction. In the Indian setting, a substantial proportion of patients with CKD are affected by anemia, which contributes to reduced functional capacity and poorer health-related quality of life [1,2]. In addition to symptoms such as fatigue, reduced exercise tolerance, poor appetite, and cognitive impairment, anemia has also been associated with progression of kidney disease and unfavorable cardiovascular outcomes [3,4].

The development of anemia in CKD is multifactorial. Reduced erythropoietin production by the diseased kidneys is the principal mechanism, but chronic inflammation, impaired iron utilization, and nutritional deficiencies also contribute to its pathogenesis [5]. Increased hepcidin activity in CKD can limit intestinal iron absorption and the mobilization of stored iron, leading to functional iron deficiency and ineffective erythropoiesis [6]. Deficiencies of folate and vitamin B12 may further aggravate the hematologic burden in these patients [7].

Conventional treatment of CKD-related anemia includes oral or intravenous (IV) iron supplementation and erythropoiesis-stimulating agents (ESAs) [8]. Although ESAs remain an important component of therapy, they require parenteral administration and may pose practical limitations related to storage, transport, and patient convenience. In addition, variable responsiveness to ESA therapy has been reported in routine clinical practice, creating a need for alternative therapeutic approaches [9].

Hypoxia-inducible factor prolyl hydroxylase inhibitors (HIF-PHIs) have emerged as a newer therapeutic class for the treatment of anemia in CKD. These agents increase endogenous erythropoietin production by stabilizing hypoxia-responsive pathways and may also improve iron metabolism [10]. Desidustat is an oral HIF-PHI approved in India for the treatment of anemia in both dialysis-dependent CKD (DD-CKD) and non-dialysis-dependent CKD (ND-CKD) patients. In phase 3 clinical studies, desidustat demonstrated non-inferiority to erythropoietin therapy in improving hemoglobin (Hb) levels in both DD-CKD and ND-CKD populations [11,12].

Despite encouraging evidence from controlled trials, real-world data on the use of desidustat in routine nephrology practice remain limited. Real-world multicenter studies are valuable because they reflect treatment outcomes in broader and more heterogeneous patient populations than those typically included in clinical trials. Therefore, the present retrospective multicenter study was undertaken to evaluate the efficacy and safety of desidustat in patients with CKD-related anemia treated across multiple centers in India.

This work was previously presented in abstract form at the European Renal Association (ERA) Congress 2025.

## Materials and methods

Study design and setting

This was a retrospective, multicenter, observational study conducted across participating nephrology centers in India. The study was coordinated across multiple tertiary care centers, including Fortis Escorts Super Speciality Hospital (Delhi), Medica Superspeciality Hospital (Kolkata), Apollo Super Speciality Hospital (Guwahati), Max Super Speciality Hospital (Noida), and Dr. Bendre Kidney Center (Meerut). Clinical records of eligible patients were reviewed over a defined study period covering cases treated between January 2024 and December 2024. All data were extracted from hospital electronic medical record databases.

Inclusion criteria

Adults aged 18 years or older with CKD-induced anemia who were prescribed desidustat and had available baseline and follow-up hemoglobin values, along with relevant clinical data, were included.

Exclusion criteria

Patients with incomplete essential clinical records, including missing baseline or follow-up hemoglobin values, CKD status, desidustat dosing details, or essential safety documentation, were excluded. Patients who received erythropoiesis-stimulating agents during the observation period; had active bleeding or major blood loss, active infection, malignancy, or other concomitant illnesses likely to affect hemoglobin response; and those lost to follow-up were also excluded.

Study population

A total of 600 patient records were screened across participating centers during the study period. After application of eligibility and evaluability criteria, 83 records were excluded, including 54 excluded at baseline, 12 with incomplete clinical records at the first visit, and 17 lost to follow-up at subsequent visits. The final analytical cohort, therefore, comprised 517 patients with CKD-associated anemia. Both dialysis-dependent CKD (DD-CKD) and non-dialysis CKD (ND-CKD; pre-dialysis) patients were included.

Sample size calculation and sampling technique

As this was a retrospective observational study, no formal sample size calculation was performed. All eligible patients meeting the inclusion criteria during the study period were included. A consecutive sampling approach was used, including all eligible patients during the study period.

Treatment protocol and follow-up

Desidustat was prescribed according to approved prescribing information and treating physician discretion. The most common regimen was 100 mg three times weekly; however, lower starting doses, including 50 mg, were used in selected patients based on clinical judgment. In dialysis-dependent patients with CKD, desidustat was administered in the early morning prior to dialysis sessions. Dose adjustments were made at the discretion of the treating clinician based on hemoglobin response and clinical judgment. Oral or intravenous iron supplementation was prescribed based on individual iron status when clinically indicated. Dosing modifications, treatment adherence, and adverse events (AEs) were recorded during follow-up visits conducted at one month, two months, and six months after treatment initiation.

Study outcomes

The primary outcome was the change in hemoglobin (Hb) levels from baseline to each follow-up visit (one month, two months, and six months). Secondary outcomes included the incidence of adverse events (AEs) and serious adverse events (SAEs), rate of treatment discontinuation, and comparison of hemoglobin response between dialysis-dependent and pre-dialysis CKD groups.

Statistical analysis

Data were analyzed using IBM SPSS Statistics version 29.0 (IBM Corp., Armonk, NY). Continuous variables were expressed as mean ± standard deviation (SD). Paired t-tests were used to compare hemoglobin levels between baseline and follow-up visits. A p-value < 0.05 was considered statistically significant. No standardized clinical scoring system or validated scale was used; outcomes were assessed using laboratory hemoglobin values and clinical record review.

Ethical considerations

The study protocol was reviewed by the applicable institutional ethics committee/authorized institutional review process at participating centers, and informed consent was waived due to the retrospective use of fully de-identified patient data.

The final analysis population differed from the preliminary conference abstract dataset following database validation and lock, which included verification of source data, exclusion of non-evaluable patients with incomplete outcome or follow-up data, and reconciliation of discrepancies across participating centers to ensure accuracy and consistency of the final multicenter dataset.

**Figure 1:**
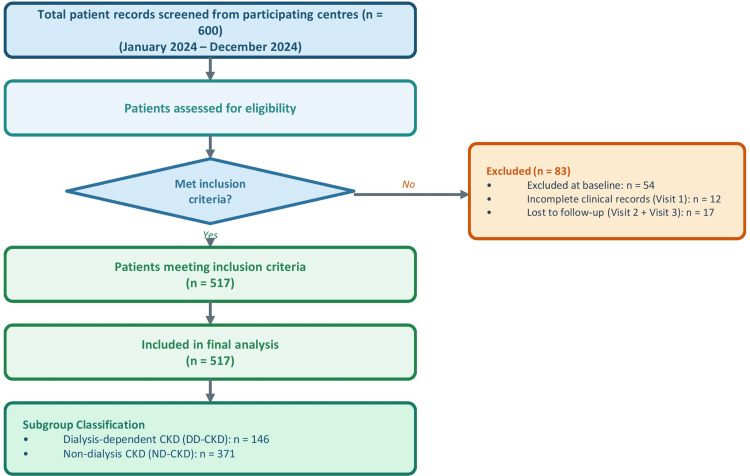
Participant selection flowchart Of 600 patient records screened across participating centers between January 2024 and December 2024, 83 were excluded due to baseline ineligibility (n = 54), incomplete clinical records at the first visit (n = 12), and loss to follow-up at subsequent visits (n = 17). A total of 517 patients were included in the final analysis and categorized into dialysis-dependent CKD (n = 146) and non-dialysis CKD (n = 371) groups. CKD: chronic kidney disease, DD-CKD: dialysis-dependent chronic kidney disease, ND-CKD: non-dialysis chronic kidney disease

The participant selection process is illustrated in Figure 1.

## Results

Among 600 screened patient records from six participating superspeciality centers, 517 patients met eligibility and evaluability criteria and were included in the final analysis. Of these, 146 (28.2%) patients were classified as dialysis‑dependent (DD‑CKD), while 371 (71.8%) patients were in the pre‑dialysis stage (ND‑CKD). The study population included 204 (39.4%) men and 313 (60.6%) women, with a mean age of 57.86 ± 14.24 years. Common comorbid conditions consisted of diabetes mellitus in 229 (44.3%) patients and hypertension in 317 (61.3%) patients. Table 1 summarizes demographic and baseline clinical features for dialysis and pre‑dialysis groups.

**Table 1:** Baseline demographic and clinical characteristics of the study population (N = 517)

Variable	Value
Dialysis-dependent	146 (28.2%)
Pre-dialysis	371 (71.8%)
Male	204 (39.4%)
Female	313 (60.6%)
Mean age (years)	57.86 ± 14.24
Diabetes mellitus	229 (44.3%)
Hypertension	317 (61.3%)

At baseline, mean hemoglobin (Hb) was 9.11 ± 1.38 g/dL overall. Baseline Hb by subgroup was 9.06 ± 1.51 g/dL in DD-CKD and 9.11 ± 1.33 g/dL in ND-CKD patients. A progressive and statistically significant rise in Hb was observed from baseline to each follow-up: baseline: 9.11 ± 1.38 g/dL, Month 1: 9.83 ± 1.52 g/dL (Δ +0.72 g/dL; +9.13%), Month 2: 10.10 ± 1.59 g/dL (Δ +0.99 g/dL; +12.05%), and Month 6: 10.13 ± 1.74 g/dL (Δ +1.02 g/dL; +12.39%). The rise in Hb is significant at all time points (p < 0.005).

Subgroup analysis of dialysis-dependent CKD (DD-CKD) and non-dialysis-dependent CKD (ND-CKD) patients demonstrated a progressive rise in hemoglobin levels from baseline, as described in Table 2. Although Hb rise was numerically greater in ND-CKD patients, the between-group difference was not statistically significant (p > 0.05). However, the rise in Hb from baseline within both DD-CKD and ND-CKD groups was statistically significant (p < 0.005).

**Table 2:** Mean Hb values at baseline and follow-up in ND-CKD and DD-CKD patients (N = 517) Hb: hemoglobin, DD-CKD: dialysis-dependent chronic kidney disease, ND-CKD: non-dialysis-dependent chronic kidney disease

Time point	Pre-dialysis patients (ND-CKD) (n = 371)	Dialysis patients (DD-CKD) (n = 146)
Baseline (g/dL)	9.11 ± 1.33	9.06 ± 1.51
Month 1 (g/dL)	9.83 ± 1.45	9.78 ± 1.69
Month 2 (g/dL)	10.10 ± 1.57	9.94 ± 1.65
Month 6 (g/dL)	10.13 ± 1.63	9.93 ± 1.90

**Figure 2:**
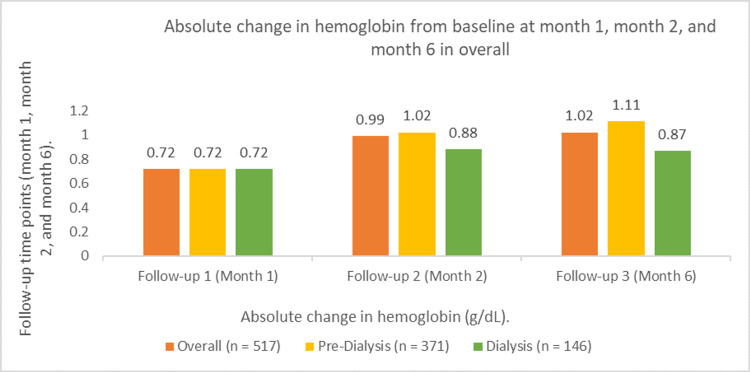
Absolute change in hemoglobin from baseline at Month 1, Month 2, and Month 6 in overall, ND-CKD, and DD-CKD groups X-axis: Follow-up time points (Month 1, Month 2, and Month 6) Y-axis: Absolute change in hemoglobin (g/dL) The figure illustrates the magnitude of hemoglobin improvement from baseline, with greater increases observed in the ND-CKD group compared to DD-CKD patients. Hb: hemoglobin, DD-CKD: dialysis-dependent chronic kidney disease, ND-CKD: non-dialysis-dependent chronic kidney disease

Overall, there was a progressive rise in Hb, with increases of +0.72 g/dL at Month 1, +0.99 g/dL at Month 2, and +1.02 g/dL at Month 6. Pre‑dialysis (ND-CKD) patients showed the most pronounced improvements compared to other subgroups in the study, reaching +1.11 g/dL by Month 6, indicating a stronger hematologic response compared to dialysis‑dependent patients. In contrast, dialysis patients experienced a more modest rise, with increases of +0.72 g/dL, +0.88 g/dL, and +0.87 g/dL at the three respective follow‑up points (Figure 2).

The majority of patients (88.18%, 456) received desidustat 100 mg three times a week (Monday/Wednesday/Friday). Oral or intravenous iron was co‑prescribed based on the iron need of the patient. Treatment adherence was clinically acceptable across follow‑ups, with no discontinuations due to drug‑related toxicity noted in the records.

Desidustat was well tolerated. Reported adverse events were mild, with constipation and throat discomfort most noted. No treatment discontinuations due to adverse drug reactions were recorded in the dataset, and no new safety signals emerged during the observation period.

## Discussion

Chronic kidney disease (CKD)-associated anemia is one of the most prevalent complications of CKD and significantly impacts patients’ quality of life, renal disease progression, and cardiovascular outcomes. Conventional treatment has traditionally relied on iron supplementation and exogenous erythropoiesis-stimulating agents (ESAs). However, ESAs require parenteral administration and strict cold-chain storage, which can limit their practicality in routine clinical settings. Desidustat, a hypoxia-inducible factor prolyl hydroxylase inhibitor (HIF-PHI), offers a novel oral therapeutic approach by stimulating endogenous erythropoietin production through stabilization of HIF pathways [13].

In this multicenter, real-world retrospective analysis of 517 patients with CKD-associated anemia, a female predominance was observed, with 313 (60.6%) women included in the study population. This differs from the phase 3 DREAM-D trial, which demonstrated a male predominance, and the DREAM-ND study, which reported a more balanced gender distribution [11,12]. Such differences likely reflect variations in real-world patient demographics compared to controlled clinical trial populations.

The present study demonstrated a progressive and statistically significant increase in hemoglobin (Hb) levels across all follow-up time points. Mean Hb increased from 9.11 ± 1.38 g/dL at baseline to 9.83 ± 1.52 g/dL at Month 1, 10.10 ± 1.59 g/dL at Month 2, and 10.13 ± 1.74 g/dL at Month 6 (p < 0.005 for all comparisons). These findings are consistent with previously published clinical trials and real-world studies evaluating desidustat, reinforcing its efficacy in the management of CKD-related anemia [11,12,14]. The sustained Hb response observed over six months suggests both effectiveness and durability of treatment.

Subgroup analysis further demonstrated that both dialysis-dependent CKD (DD-CKD) and non-dialysis CKD (ND-CKD) patients experienced significant improvements in Hb levels from baseline. However, intergroup differences were not statistically significant (p > 0.05).

The magnitude of Hb increase observed in the overall population indicates a clinically meaningful therapeutic effect. In the DD-CKD subgroup, the absolute rise in Hb was comparable to that reported in the DREAM-D study [11], suggesting that desidustat is similarly effective in dialysis-dependent patients. The trends in absolute Hb change further support consistent hematologic improvement across subgroups.

The safety and tolerability profile of desidustat observed in this study was favorable. Only mild adverse events, such as constipation and throat discomfort, were reported, and no treatment discontinuations due to drug-related toxicity were documented. Although the retrospective design may result in underreporting of adverse events, the observed safety profile aligns with previously reported clinical and real-world data [14]. The oral route of administration, along with the absence of cold-chain requirements, enhances patient convenience and may improve adherence, particularly in resource-limited or tropical settings.

The predominance of the 100 mg thrice-weekly dosing regimen (88.18%) and the use of individualized iron supplementation reflect real-world clinical practice patterns. The sustained Hb levels observed between Month 2 and Month 6 suggest that, following an initial rise, hemoglobin stabilization can be achieved with appropriate dose titration and ongoing iron optimization.

This study has several strengths. It represents one of the largest real-world, multicenter cohorts evaluating desidustat in CKD-related anemia, with 517 patients across multiple tertiary care centers in India. The inclusion of both DD-CKD and ND-CKD populations provides comprehensive insights into treatment effectiveness across different disease stages. Additionally, standardized follow-up intervals at Month 1, Month 2, and Month 6 allowed for clear evaluation of treatment response over time.

However, certain limitations must be acknowledged. The final analytical cohort was derived after exclusion of patients with incomplete records or inadequate follow-up, which may introduce selection bias inherent to retrospective analyses. The retrospective design inherently carries risks of confounding, missing data, and documentation bias. The absence of a randomized comparator group limits direct comparison with ESAs or other therapies. Additionally, inflammatory markers such as hepcidin were not evaluated because they are not routinely available in clinical practice. Patient-reported outcomes such as fatigue, exercise tolerance, appetite, and overall well-being were not systematically recorded and therefore could not be analyzed. Variability across centers, including differences in iron supplementation strategies, dialysis schedules, and dose titration practices, may also have influenced outcomes. Center-wise hemoglobin response analysis was not uniformly available across participating sites and therefore could not be systematically evaluated. Detailed cumulative oral/IV iron administration data were not uniformly available; therefore, differences in iron supplementation may have influenced Hb response. Future prospective studies with longer follow-up periods, inclusion of cardiovascular and thromboembolic safety endpoints, and assessment of patient-reported outcomes are warranted to further define the role of desidustat in the management of CKD-related anemia.

## Conclusions

In this large real-world multicenter cohort, desidustat was associated with sustained and statistically significant improvement in hemoglobin levels over six months in patients with CKD-related anemia, with a favorable tolerability profile and no new safety signals. These findings support desidustat as a practical oral treatment option for CKD-related anemia in routine nephrology practice. Prospective controlled studies with standardized iron assessment and patient-reported outcomes are warranted.
